# Overcoming the myths of esketamine administration: different and not difficult

**DOI:** 10.3389/fpsyt.2023.1279657

**Published:** 2023-11-23

**Authors:** Florian Buchmayer, Siegfried Kasper

**Affiliations:** ^1^Department of Psychiatry and Psychotherapy, Hospital of Brothers of Saint John of God, Eisenstadt, Austria; ^2^Center for Brain Research, Medical University Vienna, Vienna, Austria

**Keywords:** esketamine, treatment-resistant, depression, intranasal, TRD (treatment-resistant depression), MDD (major depressive disorder)

## Abstract

Intranasal esketamine for treatment-resistant depression has been introduced and approved by the FDA and EMA in 2019 and 2020, respectively. Since then, the administration practices were found different among countries. Major depression has a high impact on many humans lives worldwide and more than a third of treated people are not responding after several treatment attempts. Additional administration with esketamine closed this gap for more than the half of these non-responders. Guidelines for the treatment of major depression recommend starting with add-on esketamine after 2–4 serious attempts of treatment with standard antidepressants (SSRI/SNRI) irrespective of augmentation with others, e.g., second generation antipsychotics or lithium. Thus, intranasal esketamine became an important role in the evidence-based treatment of major depression. The authors review and critically evaluated published articles focusing on preparation, management and observation of intranasal esketamine treatment. There exists a clear recommendation for administrating intranasal esketamine in a medical environment, not limited to a clinical setting for selecting the dose, monitoring the improvements and managing adverse events. The administration of intranasal esketamine is considered as safe during the application itself and long-lasting or severe adverse events during long-term treatment are very rare. Since this is a new approach for treatment application psychiatrists face new different but not difficult treatment procedures compared to prescribing only a medication.

## 1 Introduction

Traditional monoamine-based antidepressants fail in about one third of the population to achieve full recovery in individuals with major depressive disorder. Also manual-based psychotherapy, such as cognitive-behavioral therapy, has limited efficacy as a monotherapy for treatment-resistant depression (TRD) (1, 2). The practical definition of TRD is “the failure of at least two different treatments with antidepressants of adequate duration and dose in the current moderate-to-severe depressive episode” (3). Although health regulatory authorities use this definition for granting indications there is still an ongoing scientific debate upon a clinical definition for this condition. However, TRD is influenced by various neurobiological (HPA-axis, inflammatory response, BDNF), genetic (5-HT), and clinical factors (comorbidities) (4). While the approval of ketamine and esketamine for TRD has opened up new possibilities for patients, concerns have been raised regarding its long-term efficacy, safety, tolerability, patient selection, and the risk of substance use disorder. Due to the increasing number of practitioners and clinics providing ketamine treatments, it is crucial to have a comprehensive grasp of these domains, as elucidated by a collective of international researchers specializing in this field (5). Cardiovascular effects are an important safety consideration when using ketamine and esketamine. Ketamine has been associated with transient cardiovascular stimulatory effects, including an increase in blood pressure and heart rate. This poses potential challenges in the treatment of TRD patients who often have comorbid hypertension and cardiovascular disease. The cardiovascular effects of esketamine nasal spray have been evaluated in a large cohort of TRD patients within the esketamine clinical development program to better understand its impact on this patient population and they found that these blood pressure elevations are generally transient, asymptomatic, and not associated with serious cardiovascular safety sequalae (6).

There is evidence, that conditions such as obesity, type 2 diabetes mellitus, and metabolic syndrome, which are associated with insulin resistance, are associated with prolonged treatment durations and are linked to treatment resistance in major depression. While esketamine treatment does not lead to these comorbidities, they are known side effects of several other antidepressant medications (7–9).

Furthermore, the dissociative effects of ketamine and esketamine have been observed in clinical trials. Interestingly, there is a discussion in the literature regarding the relationship between dissociation and subsequent antidepressant effects. However, based on the development program sent to the health authorities in US and Europe it does not seem that there is a significant correlation between the appearance of dissociation and improvement in depressive symptoms (10, 11).

Some psychiatrists still hesitate to use esketamine as treatment and while esketamine is going to change the treatment of depression fundamentally, this article focus on the following main issues: selection of patients, administration of esketamine, safety and risk management as well as long-term effects.

### 1.1 Indications for intranasal esketamine treatment

**Figure 1:**
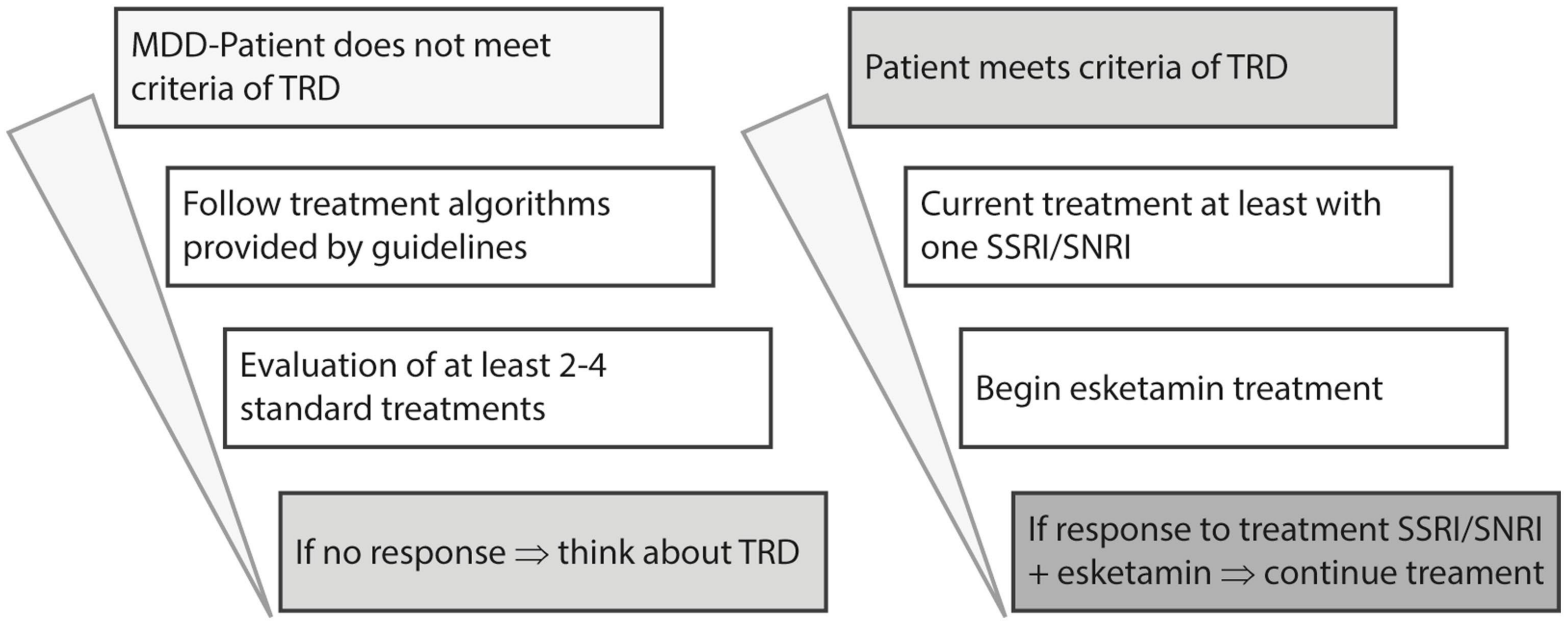
Modified algorithm of pharmacological MDD treatment, based on Kasper et al. (4).

Up to one third of people suffering from MDD (major depressive disorder) do not respond to several treatment attempts. Terms like treatment-resistant or difficult to treat are used to describe the course of illness within the diagnose. Although various treatment algorithms have been developed in the past for the treatment of MDD, the most current recommendation states that in the first and second lines of treatment, two SSRIs/SNRIs should be used, with options for augmentation (second-generation antipsychotics or lithium), dose escalation (after measurement of plasma levels), or combination of antidepressants considered. If there is no response the diagnose of TRD can be established and a third line treatment with esketamine can be considered (4, 12) (see Figure 1).

Apart from its approval for treating TRD, esketamine is recommended for use in cases of acute, moderate to severe depression, whether or not suicide ideation is present. Several reports suggest that esketamine may be effective in reducing suicide ideation, although findings vary among different studies since it is not easy to disentangle the antidepressant from the anti-suicidal effect which usually are reduced parallel and therefore there is no room for differentiation (13–15).

### 1.2 Efficacy of intranasal esketamine

The proposed clinical effects of ketamine and its mechanism of action in treatment-resistant depression (TRD) involve various pharmacodynamic targets. Esketamine is discussed to promote synaptogenesis and synaptic potentiation by binding to the phencyclidine site of the N-methyl-D-aspartate receptor (NMDAR), leading to a glutamate surge. This surge activates the AMPA receptor, initiating intracellular signaling cascades that increase brain-derived neurotrophic factor (BDNF) activity. Esketamine may also affect the subgenual anterior cingulate cortex (sgACC), reducing overactivity associated with depression. Additionally, esketamine may interact with opioid receptors, although further research is needed to understand its role, but interestingly it is thought that analgesic effects are not provided along with this mechanism (4, 5, 16, 17).

Esketamine has a plasma protein binding of approximately 43%–45%, thus is a lipophilic substance. The elimination half-live is 7 to 12 hours for esketamine. Intranasal esketamine has a bioavailability of about 30%–50% and is metabolized mainly by CYP3A4 and CYP2B6 enzymes (18).

The rapid and robust antidepressant effects of esketamine have been observed in patients with TRD within 4 weeks. Multiple studies have demonstrated the efficacy of esketamine as an adjunctive treatment alongside an oral antidepressant. Additionally, long-term maintenance studies have shown sustained antidepressant effects and delayed relapse with esketamine. After an induction phase of 4 weeks a response rate of 78,4% and a remission rate of 47,2% was observed. During the maintenance phase the remission rate increased up to 58,2% (19–22).

In patients older then 64 years the efficacy of esketamine treatment may be limited (23).

### 1.3 Adverse events of intranasal esketamine

In clinical studies, esketamine nasal spray adverse events followed a similar time course: within the first 10 to 40 min after administration typically AEs (adverse events) appear and 60 to 90 min later most of them were alleviated or resolved. From session to session they get more moderate or do not appear any more (6, 19, 21, 24). Elevation of blood-pressure is an AE which should be monitored during the session to recognize an eventual hypertensive crisis. Elevations are usually mild (systolic differences did not increase 15 mmHg) and transient (21).

Esketamin can elicit dissociative effects which are generally known as psychoactive or as psychodelic effects. They include several effects like perceptual (auditory, visual, proprioceptive), detachment from body or from self or the external world, tranquility and openness feeling peace and calm, mystical-type-effects feeling unity and relativeness or spiritual. Although fear and anxiety could appear during the session (25). Analyses of the Transform-2 study did not show any correlation between dissociative and antidepressant effects (11).

These esketamine-induced phenomena occur in about one fourth of patients and are transient and very subjective and variable in intensity and content. It seems that some of these effects are dependent from the patients mood an experiences in the hours before the session is started, therefore it seems to be wise to ask the patient to think about positive or neutral events, rather than negative or problematic life circumstances.

Sedation can occur as an AE in the treatment with esketamine and therefore checks of vital signs are recommended (21). Nausea, dizziness, vertigo and somnolence are common during the treatment. Several other AE have been observed during the treatmtent sessions with esketamine: dysgeusia with a bad, metallic or bitter taste, nausea, vomiting. Some patients react with euphoric mood or feel drunk (3, 24) (see Table 1).

**Table 1:** Most common adverse events during intranasal esketamine treatment (3).

Adverse events (AE)	%
Dizziness	31
Dissociation	27
Nausea	27
Headache	23
Somnolence	18
Dysgeusia	18
Vertigo	16
Hypoaesthesia	11
Vomiting	11
Blood pressure increased	10

In general, type and frequency of AEs are consistent in all age groups (23). Symptoms like fatigue or insomnia may occur in about one fifth of patients who discontinue the treatment in or after the maintenance phase (21).

Compared to other pharmacological antidepressant treatments these side effects are short lasting and do not pose a problem for the patient in the long-term treatment.

### 1.4 Contraindications

Contraindications are summarized in the SmPC for intranasal esketamine SPRAVATO^®^ and are listed in the following: hypersensitivity to (es)ketamine or other compounds, untreated or suboptimal treated arterial hypertension, elevation of intracranial pressure, normal pressure hydrocephalus, acute cardiovascular events, coronary heart disease, intracerebral hemorrhage and stroke (3).

Relative contraindications are suicidal thoughts and/or behavior, acute psychosis, acute or florid drug-abuse, neurological impairments, respiratory depression, unstable cardiovascular disease, severe hepatic impairment, pregnancy, breast-feeding.

## 2 Methods

The authors reviewed and critically evaluated published articles in Pubmed/NLM/NIH (keywords: intranasal, esketamine, depression) from 01/2012 to 04/2023. The primary goal for the research was to give a clear overview about intranasal esketamine treatment focusing on an implementation in practice. Therefore 86 clinical trials and safety reviews were screened for relevant information according to the section topics (treatment setting, patient selection, personnel, treatment plan, dosing, documentation, AEs and management, end of treatment). The authors are well trained in the application and management of intranasal esketamine treatment and thus try to give useful recommendations and offer some practical help for the management of TRD-patients. With this article the authors want to address physicians and psychiatrists working in an outpatient environment.

## 3 Implementation of intranasal esketamine treatment

### 3.1 Preparation

Risk management in psychiatry involves crucial steps: identifying risks, preventing known risks, and devising plans to mitigate potential harm if risks materialize. Common risks include suicide, drug abuse, relapse and violence towards others, and patient safety concerns like adverse effects. In medical practice, standards such as risk/benefit analyses, shared decision-making, informed consent, treatment planning, and thorough documentation and working with checklists are integral components that all physicians are trained to implement (26–28).

Even esketamine is known to be a safe compound and is lacking respiratory depressive effects, there are mild adverse effects occurring in about two thirds of patients and very seldom severe AEs. During the esketamine treatment evidence-based strategies can be used to compensate for these AEs.

Every physician using esketamine treatment should be well prepared and organized in several aspects, shown in the following sections.

#### 3.1.1 Patient selection

To be eligible for treatment, patients must meet the indication criterion of having treatment-resistant major depression, and contraindications must be ruled out. Individuals with a short history of depression should undergo a comprehensive medical and psychiatric evaluation to identify any physical causes or comorbidities. Extra attention should be given to patients with substance dependency, severe personality disorders (specifically of the borderline type), non-schizophrenic psychotic episodes, post-traumatic stress disorder, or other significant traumatic experiences (4). Treatment with esketamine appears suitable for individuals with treatment-resistant depression and comorbidities who find themselves in a primarily stable, abstinent, and non-psychotic phase of their condition. This choice is guided by considerations of patient compliance and the need to minimize potential side effects, without intending to preclude these patient groups from consideration.

Anxiety symptoms or anxiety disorders which are frequent in TRD patients do not influence the efficacy of the antidepressant esketamine effect (29).

Suicide ideation, acute or in the past, with or without suicide attempts, is not an exclusion criterion, but patients with acute suicide ideation should be considered to send to clinic (4) and monitored accordingly like every patient with this illness characteristic.

#### 3.1.2 Setting

##### 3.1.2.1 Treatment room

**Figure 2:**
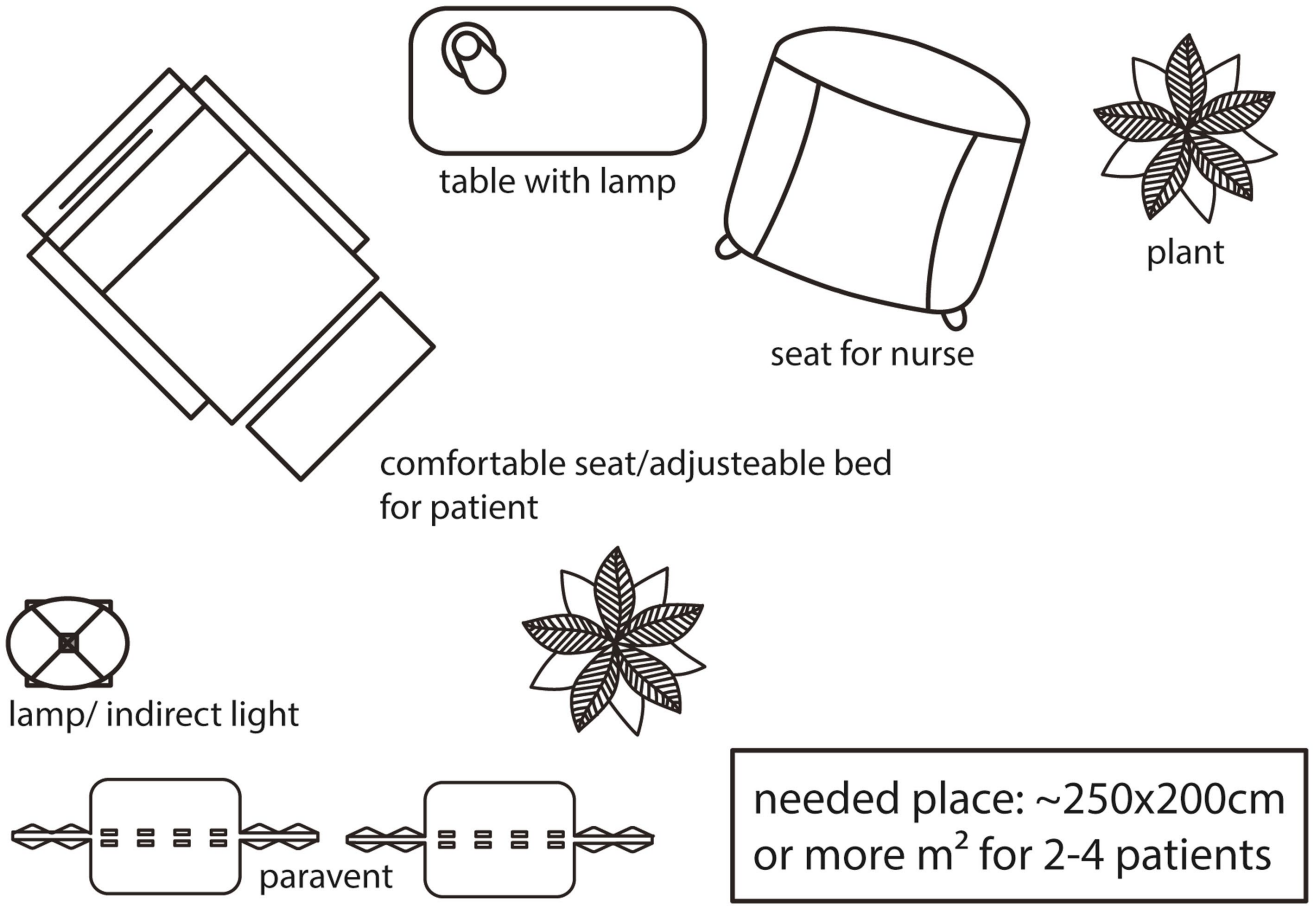
Visualization of a treatment room in 2D from bird’s eye view. Instead of seats, there can be adjustable beds - more than one seat/bed in a bigger room is also feasable.

When administering esketamine treatment, it is essential to select a calm and isolated room. A comfortable seat or an adjustable bed should be used, and a room of adequate size should be provided if more than one seat or bed is required. Many patients experience heightened sensitivity to sensory stimuli during esketamine’s influence, which could potentially disrupt their thoughts. Thus, it is crucial to have reduced light, sound, and traffic in the room. Some patients may feel particularly vulnerable, making a single room preferable to prevent overarousal or adverse reactions. From the physicians’ standpoint, this room should be spacious enough to accommodate patients comfortably and facilitate medication administration while also allowing for effective management of any potential adverse effects. Installation of a communication-system helps patients to call for help or assistance and provides additional safety (4) (see Figure 2).

##### 3.1.2.2 Patients and music

It could be beneficial to suggest that patients listen to meditative and soothing music (using headphones) during their treatment sessions. This may assist in reducing negative dissociative effects, anxiety, and confusion, and potentially foster a positive experience (30).

##### 3.1.2.3 Planning treatment sessions

Each esketamine treatment session typically spans from 1.5 to 2 h in duration. The effects of the treatment peak within the initial 10 to 20 min and usually taper off after approximately 60 min. Patients should be advised to set aside roughly 2 h for each session to allow for a fulfilling experience. Following a session, patients should refrain from driving, as their concentration may be compromised, and work performance might be hindered for several hours. Certain patients might require assistance with transportation. During the initial four-week induction phase of esketamine treatment, it is imperative to schedule two sessions per week. Following this phase, sessions should continue at a frequency of once a week for a minimum of an additional 4 to 8 weeks. A reassessment to potentially adjust the frequency of treatment sessions, transitioning from once to twice a week, should be conducted after 8 to 12 weeks (3, 13).

##### 3.1.2.4 Improving compliance

Enhancing patient adherence to a treatment regimen requires the implementation of several measures according to shared decision making. One approach to aid in treatment decision-making is through a shared decision model, comprising the following three sequential phases: 1. Choice talk: offer choices, check reaction, check preferences, justify choice. 2. Option talk: check knowledge, list and describe options including harm and benefit, provide decision support, summarize. 3. Decision talk: focus on preferences, elicit a preference including a back-up plan, move to a decision, offer review (31).

The assessment of benefits and risks should encompass a view of treatment outcomes, engagement in sessions, individual adverse effects and their handling, assessment intervals, and treatment duration. Having a back-up plan could play a pivotal role in decision-making, as patients might anticipate recovery and so alternative choices should be made available. Having professional staff present during sessions, especially when patients are encountering adverse effects, can be reassuring and instill a sense of security. Conducting brief assessments of mental shifts and the progression of particular effects or challenges could enhance the patient-professional relationship (32).

#### 3.1.3 Personnel

A universal guideline, subject to customization according to each country’s legal framework, is that healthcare professionals (including physicians and medical staff) should receive specialized training to effectively engage with psychiatric patients and deliver treatment in an outpatient environment. The treatment process should encompass preparation, administration, observation, and discharge, forming integral components of the treatment session routine. While it may not be mandatory for a professional to be constantly present in the treatment room, this could be considered as required, at least on-demand. Professionals should also undergo training in life-support techniques, and an emergency kit should be readily available within or near the treatment area. In situations where patients require assistance due to adverse events a well-defined plan outlining the professional’s response should be communicated within the team.

### 3.2 Treatment administration

Obtaining the patient’s informed consent is crucial before the initial administration of esketamine. Furthermore, patients should receive prior instructions to refrain from eating for 2 h before esketamine administration, avoiding the use of decongestant nasal drops 1 h before, and abstaining from consuming beverages 30 min before the treatment. Prior to each session, blood pressure should be assessed and if it is elevated (>140/90mmHg for patients 18-64 years old or > 150/90mmHg for patients 65 and more years old) appropriate measures should be taken to reduce/normalize blood pressure before starting the treatment. After patients received detailed instructions by the professional, they are responsible for self-administering the esketamine nasal spray. There should be an interval of 5 min between the application of two sprays.

#### 3.2.1 Dosing

**Figure 3:**
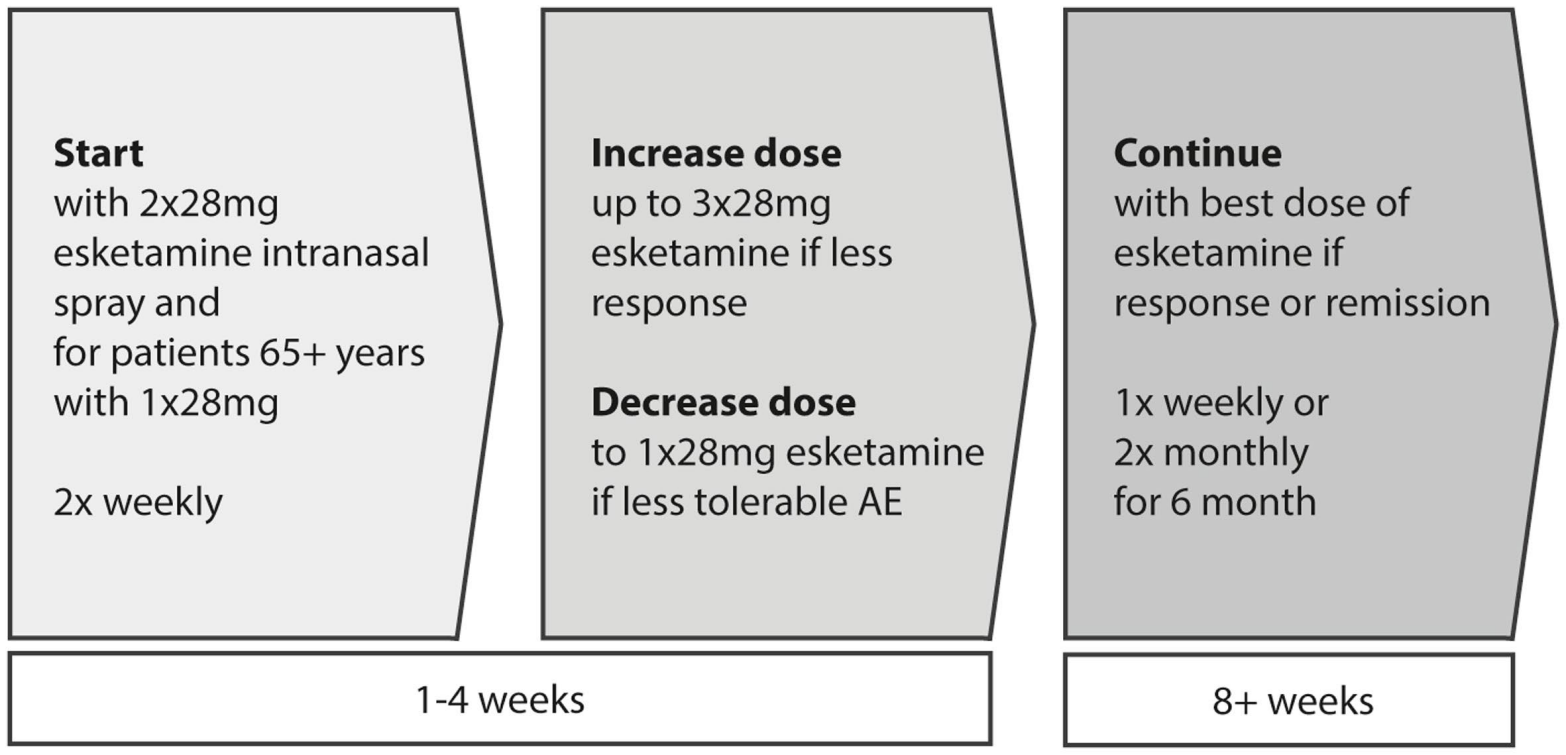
Induction (first 4 weeks) and maintenance (afterwards) phase in intranasal esketamine treatment. For patients aged 65 or above the first dose should be 28 mg.

At the start, esketamine intranasal (i.n.) is given at a dose of 56 mg per application, with 65-year-old and older patients receiving 28 mg initially. Starting from the second application, those under 65 years may receive either 56 mg or 84 mg per application, while patients aged 65 and above may receive 28 mg or 56 mg per application. The dose can be maintained or increased to 84 mg based on individual response and tolerance during subsequent treatments. During the induction phase lasting four weeks, esketamine i.n. is administered twice weekly. At the latest by the end of this phase, the treatment’s effectiveness is evaluated to determine whether it should continue. If there’s a suitable therapeutic response and good tolerance, the frequency of esketamine i.n. administration is reduced to once weekly starting from the fifth week for maintenance therapy. Further reduction to once every two weeks can be considered from the ninth week onwards. It is advisable to continue treatment for at least 6 months after an improvement in depressive symptoms has been observed (see Figure 3).

Clinically, dose selection for esketamine treatment should prioritize the antidepressant response and the balance between effectiveness and tolerability, rather than emphasizing the induction of dissociative symptoms to confirm therapeutic adequacy (11).

#### 3.2.2 Observation

Throughout treatment sessions, patients’ blood pressure should be measured on at least two occasions – before the session’s commencement and approximately 30–40 min thereafter. It is advisable to provide company and closely monitor for any adverse events during the initial session. In the periods between treatment sessions, adherence to the recommendations involves the ongoing assessment of treatment efficacy. To facilitate this, a range of psychometric scales are at one’s disposal. Among the most highly recommended options are MADRS and PHQ-9, though HAMD-17 and IDS-30 (Montgomery-Åsberg Depression Rating Scale, Patient Health Questionnaire, Hamilton Depression Rating Scale, Inventory of Depressive Symptomatology) also appear to be suitable choices.

#### 3.2.3 Overall treatment plan

Maintaining an ongoing assessment of the antidepressant effects of esketamine treatment is of utmost importance. Following the initial induction phase lasting four weeks, the therapeutic benefit should be evaluated to determine need for continued treatment. Several clinician-rated and self-reported scales can be administered for this purpose, e.g., MADRS, or PHQ-9. Approximately 12 weeks following this initial evaluation, a subsequent assessment of the maintenance phase’s effectiveness is advisable. While periodic evaluations of adverse events are necessary, most of the treatment emergent adverse events are primarily confined to the treatment session (in detail, see below). In any phase of the treatment particular attention should be directed towards any sign of suicidal ideation, or impending relapse, as it generally advised in guidelines when treating TRD patients (27).

For long-term treatment (more than 1 year), patients and physicians can anticipate that adverse effects will remain comparable to those experienced during the initial year, and that positive effects such as a reduced suicide rate or decreased global impairment continue to occur in these patients (33, 34).

Recommendations for the duration of esketamine treatment range from 6 months to 1 year and more, contingent on the observed antidepressant response. Several approaches can be considered. One approach involves concluding treatment after 6 months of maintenance, with mental assessments conducted every 4 weeks. Alternatively, treatment duration can be influenced by patient-reported improvements, measured additionally by the number of good days, leading to adjustments in treatment intervals based on these reports. When intervals reach 2 weeks, treatment discontinuation after at least 8 weeks might be considered. Another option involves terminating treatment between 6 to 12 months, with intervals extended to 2 weeks after 8 weeks of maintenance by standard. It is crucial to determine the stability of the mental state post-treatment discontinuation to prevent relapses, thereby warranting the reinitiation of esketamine treatment.

Instances where patients miss treatment sessions due to illness or vacation require evaluation by professionals. Any observed reduction in efficacy should prompt a temporary shift to once or twice-weekly intervals for 1–2 weeks, until the previous effect is reestablished.

For cases of relapse during esketamine treatment, several options can be explored for remission. Dose escalation, interval adjustments, or a combination of both (booster sessions) should be considered. Evaluating oral antidepressant medication (also by plasma levels, or if possible also with determination of Cytochrom enzymes) is recommended, though discontinuing oral antidepressants during a successful esketamine treatment is not recommended.

Some patients may experience a diminishing antidepressant effect during esketamine treatment, stemming from various causes. Efforts to boost the treatment’s effectiveness can be attempted. If unsuccessful, discontinuation of esketamine treatment is advisable, necessitating consideration of alternative antidepressant treatment strategies, like electroconvulsive treatment (ECT).

While there is currently no evidence-based guideline for the management of anomalies of the treatment plan, the authors find it appropriate to consider such a course of action.

#### 3.2.4 Evidence based management of AEs

The prevalent adverse events are prone to emerge shortly following the administration of esketamine and typically resolved within 60–90 min. Generally, these instances do not necessitate specific interventions (24).

Prophylactic treatment with antiemetics such as ondansetron may help mitigate nausea and vomiting. Some fruit punch-flavored powdered drink mix or some bon-bons could attenuate dysgeusia and probably reduce nausea (35, 36).

Hypertensive emergency may be considered an extremely rare event during esketamine treatment. Elevated blood pressures that do not meet the criteria for hypertensive emergency (more than 180/110 mmHg and presence of end-organ indicators) should not be treated with antihypertensive agents.

Furthermore, it is imperative to observe patients for concerning indicators, such as intense chest pressure or pain, sudden and severe difficulty in breathing, acute and severe abdominal pain, or a notable reduction in consciousness level. In instances like these, potential vascular incidents like acute coronary syndrome, aortic dissection, or stroke should be taken into account. Maintaining vigilant monitoring and regulation of blood pressure is crucial, and arrangements should be made for immediate transportation to the emergency department. For example, uradipil (12,5 – 25 mg i.v.) or nitroglycerine (0,4–1,2 mg s.l.) are evidence based antihypertensive treatment options with rapid onset (37, 38).

Sedation, which could be measured by using the Modified Observer’s Assessment of Alertness and Sedation (MOAA/S) score, is seldom deep or meets the criteria for a light general anesthesia (scores of 1 and below). Nevertheless, patients who underwent profound sedation did not necessitate the need for ventilatory support or resuscitation, and they regained consciousness spontaneously. It is advisable to assess essential indicators, such as blood pressure and oxygen saturation, during this period (21).

Dissociative effects typically manifest as temporary and mild adverse reactions, with a duration of approximately 1 to 1.5 h. In cases where the dissociative effects become highly intense, potentially involving distressing or unsettling content, this is commonly referred to as a “bad trip.” This experience might resemble a brief psychotic episode. To minimize the likelihood of such occurrences, it is recommended to avoid any sources of stress or troubling thoughts before embarking on the esketamine session. Should a patient already be undergoing a “bad trip,” it is advised to provide companionship while employing a slow and composed speaking style. If necessary, a pharmacological intervention involving a benzodiazepine, such as 5–10 mg of diazepam or 1–2 mg of lorazepam administered intramuscularly or intravenously, may be warranted. Following the alleviation or resolution of symptoms, allocating additional time to discuss the experience is important, reassuring the patient that the events were akin to a dream and not based in reality. Prior to the subsequent administration of esketamine, a brief feedback session coupled with reassuring words should be conducted to help alleviate the patient’s anxiety (4). If there is an individual request for accompanying psychotherapy before or after the treatment sessions, patient needs could be supported. This may help to increase compliance and could be beneficial for the treatment-outcome.

Ordinarily, the presence of a physician or nurse can help alleviate panic attacks. If this measure appears inadequate, an oral benzodiazepine, such as diazepam (5–10 mg) or lorazepam (1–2.5 mg), can be administered. To preempt panic attacks during sessions, conventional approaches for managing anxiety disorders, like relaxation techniques or psychotherapy, can be employed. In instances of recurring and enduring panic attacks, it might be prudent to review the current medication regimen and opt for a premedication strategy involving anxiolytic properties (39).

Suicidal thoughts and suicide attempts are recognized as potential risks associated with depression and any form of antidepressant treatment. Multiple international and national directives for suicide prevention are available for reference. It is advised to conduct regular screenings for suicidal thoughts and attempts, tailored to the treatment’s progression, and to be particularly vigilant regarding instances of self-harming behavior (40–42). Since TRD is a severe illness patients should be monitored accordingly throughout the treatment procedure (see Table 2).

**Table 2:** Summary of specific treatment of adverse events during intranasal esketamine treatment.

AE	Specific treatment
Nausea, vomiting	Antiemetic, e.g., ondansetron
Hypertensive emergency	Antihypertensive, e.g., uradipil or nitroglycerine
Cardial emergency	e.g. resucitation, call emergency hotline
Deep sedation	Check vital paramters and signs, if ok: wait
Intense dissociation, “bad trip”	Personnel reassuring presence, administration of anxiolytics or tranquillizers, e.g., Lorazepam or diazepam
Panic attack	Personnel reassuring presence, administration of anxiolytics or tranquillizers, e.g., lorazepam or diazepam
Increased suicide risk	Follow international/national guidelines, think about for admission to clinic, administration of medication, do not leave patient alone

#### 3.2.5 Documentation

Maintaining physician documentation is obligatory, influenced by legal considerations that differ across countries. In all circumstances, it is advisable to consolidate the outlined suggestions within this article and furnish an illustrative instance of tailored documentation pertinent to esketamine treatment.

### 3.3 End of treatment

Assessment for discontinuing esketamine antidepressant treatment should occur after a 4-week induction phase and, at a minimum, following 6 months of maintenance. In some instances, extended treatment lasting several years (more than 1 year) may be required to sustain the antidepressant effect. If the treatment proves ineffective, discontinuation can be considered at any point. Additionally, patients might discontinue treatment due to intolerable adverse effects, alterations in scheduling, or other factors.

It is advisable to cease treatment during a stable phase of response or remission. Upon concluding esketamine treatment, individuals may experience an increase in symptoms or the potential for relapse. While there is currently no evidence-based guideline for resuming treatment in the event of relapse or worsening symptoms, the authors find it appropriate to consider such a course of action.

## 4 Conclusion

Intranasal esketamine, administered alongside an SSRI/SNRI, proves to be a safe and effective remedy for individuals grappling with treatment-resistant depression. In patients with Treatment-Resistant Depression (TRD), esketamine demonstrates significant superiority over extended-release quetiapine (quetiapine XR) as an adjunctive treatment in terms of response, remission, and relapse prevention over a period of 32 weeks (43). The treatment is different to just prescribing an antidepressant pill, but not difficult, like for instance ECT. This treatment approach boasts a swift onset, thanks to its brief half-life, and is associated with infrequent short-lived adverse effects during its application intervals. The treatment session itself spans 90 to 120 min. Following an initial 4-week induction phase comprising 8 sessions, the first assessment of efficacy, typically utilizing standardized metrics such as MADRS, should be conducted. In the presence of positive outcomes, the treatment should be sustained for around 6 to 9 months, entailing 2 or 4 sessions per month. As the number of sessions increases, the occurrence of adverse effects generally diminishes. Adverse effects like elevated blood pressure, dissociation, nausea, somnolence/sedation, and panic attacks necessitate careful medical attention. Instances of hypertensive crisis requiring intervention are exceedingly uncommon (see Table 3).

**Table 3:** Summarized overview of important facts for an esketamine treatment.

Condition	Preferable	With caution
Patient selection	Diagnosed with TRD or acute and severe MDD Running on SSRI/SNRI Good compliance	Substance-use disorder Borderline personality disorder Anxiety/panic attacks Suicide ideation Unstable cardiovascular disease Somatic multimorbid patients
Treatment setting	Calm/isolated room Observation and call-button Patient can listen to music Talk to patient bevor and after session	Loud noises Less trained personnel Unpleasent odors Not reachable personnel on call
Treatment plan	Clear structured treatment plan and sessions Induction phase 8 sessions/m Maintenance phase 2–4 sessions/m Doses ranging from 28 to 84 mg per session	Interruptions caused by: Problems to provide a continuous treatment Problems with health insurance companies
AE management	Talking to the patient Administration of benzodiazepines, antiemetic and antihypertonic drugs Resuscitation, emergency call	Treating more than 2 patients at the same time could get challenging for the personnel
Documentation	Screening and evaluation (MADRS) Short docu for every session	Any problems with docu

To achieve a favorable treatment outcome, certain conditions or guidelines are condensed into recommendations labeled as either “preferable” or to be approached “with caution.” Physicians should pay attention to patients or circumstances when any items in the “with caution” column are evident. Patients with contraindications should refrain from receiving esketamine treatment.

For physicians offering intranasal esketamine treatment, the utilization of checklists, predefined documentation, and a concise adverse event management plan are pivotal. Moreover, involving the patient in a shared decision-making process and consistently evaluating treatment progress contribute to the patient’s sense of security throughout the treatment trajectory. This article is designed to assist physicians in extending intranasal esketamine treatment to their patients, affording them a substantial opportunity to recover from treatment-resistant and chronic depression.

## Author contributions

FB: Conceptualization, Visualization, Writing – original draft, Writing – review & editing. SK: Conceptualization, Writing – review & editing.
