# Supplementary material for: Overcoming the myths of esketamine administration: different and not difficult
Source: Front Psychiatry. 2023 Nov 23;14:1279657. doi: 10.3389/fpsyt.2023.1279657 (PMC10701548; doi:10.3389/fpsyt.2023.1279657)
Supplement: Supplementary file 1 [file Table_1.DOCX]

**Additional Documentation for Esketamine Treatment**

| Date and time of application: | |  | |
| --- | --- | --- | --- |
| Esketamine dose and application interval: | | Ch.-B.: | |
| Report of patient: | | | |
| AE and course: | | Intervention: NO  - if other specify: | |
| Risk of suicide: NO - LOW - HIGH  - or specify: | | General physical condition: GOOD  - if other specify: | |
| 1. blood pressure measurement (0min): | | / mmHg /min | |
| 2. blood pressure measurement (40min): | | / mmHg /min | |
| Notes: | | | |
| MADRS / HAMD-17: | PHQ-9 / IDS30-SR: | | MOAA/S / GCS: |
| Other tests: | | | |

**If not otherwise documented:**

- Diagnoses:
- Current oral / parenteral medication:
- Psychotherapy:
- Medical / treatment history: substances, dosage, duration, treatment-strategies
- Medical reports
- Allergy/hypersensitivty: no -- if other specify:
- Indication for esketamine treatment: # of antidepressant treatments, TRD
- Contraindications for Spravato: no -- if other specify:
- Informed consent for esketamine treatment: j/n

**How to use this template**

Fill out before the session:

- Report of patient
- First blood pressure measurement
- Test results

Fill out all other fields during or after the session.

Describe the AEs and their intensity. If the course of AE’s need some intervention, specify it in this field.

Stage the risk of suicide and the general physical condition at the end of the session, before your patient leaves, and mark it.

Use the notes field for your reminders, marks, summaries and others.

Print it or integrate it in your software.
